# Relationship between Body Mass Index and Tooth Decay in a Population of 3–6-Year-Old Children in Iran

**DOI:** 10.1155/2015/126530

**Published:** 2015-02-18

**Authors:** Leila Shafie Bafti, Maryam Alsadat Hashemipour, Hamidreza Poureslami, Zeinab Hoseinian

**Affiliations:** ^1^Department of Pediatric Dentistry and Kerman Oral and Dental Disease Research Center, Kerman Dental School, Kerman 7618759689, Iran; ^2^Kerman Oral and Dental Disease Research Center, Kerman Dental School, Kerman 7618759689, Iran; ^3^Department of Pediatric Dentistry, Bandar Abbas Dental School, Bandar Abbas, Iran

## Abstract

The aim of the present study was to evaluate the relationship between BMI and tooth decay in a population of Iranian children. In this cross-sectional descriptive/analytical study, 1482 children were selected from kindergartens and preschool centers in Kerman, Iran. The children underwent examination of deciduous teeth (using the dmft index) after determination of height and weight for calculation of BMI. The relationship between BMI (after adjustment for age) and dmft was determined using Poisson's regression model. The mean of dmft in children with normal BMI was 1.5-fold that in subjects with extra body weight. Age had a significant effect on dmft. In addition, dmft was higher in boys compared to girls. The results of the present study showed that caries rate in the deciduous teeth of 3–6-year-old children decreases with an increase in body weight.

## 1. Introduction

Obesity is the result of chronic inconsistency between the intake and expenditure of energy. Studies have shown that the majority of children are overweight due to the intake of low-density foods instead of fruits and vegetables and also due to high-fat and high-carbohydrate foods and a sedentary life style; in addition, some socioeconomic factors are involved. Obesity consists of an exceedingly complex group of diseases and probably should be characterized as a syndrome. The etiologies that contribute to obesity and can be modified by physicians include dietary and exercise patterns, endocrine and metabolic diseases, genetic factors, environmental factors, familial and ethnic factors, stress, drugs, and abnormal regulation of body weight or body fat [1–4]. During the past 15 years, there has been an ever-increasing trend in weight gain in Iranian children [5]. Properly controlled studies have shown a relationship between diet and dental caries. It appears that common risk factors such as intake of foods rich in carbohydrates influence the incidence of tooth decay and obesity [3–5]. It is clear that some dietary habits or malnutrition affects caries rate due to the irrefutable effects on the secretion and composition of saliva [5–7]. Presence of confounding variables such as diet or the individual's socioeconomic status or background variables, including age, personal hygiene, and the amount of fluoride in the diet, has resulted in an inability to express a firm opinion in the regard. Several studies to date have evaluated the relationship between tooth decay and obesity; however, the results have always been different and sometimes contradictory [8–14].

To our knowledge no studies have so far been carried out in this respect in Iranian preschool children and also since there are contradictions in the results of studies carried out elsewhere in the world, the present study was undertaken to evaluate the relationship between the deciduous tooth decay and BMI in a population of 3–6-year-old Iranian children.

## 2. Materials and Methods

The present cross-sectional descriptive/analytical study was carried out by clinical examination and the questionnaires were completed by an examiner. The subjects consisted of 3–6-year-old children in 23 kindergartens and preschool centers in Kerman, south east of Iran, in 2009-2010. Data were collected through clinical oral examinations, anthropometric measures, and structured questionnaires in order to assess socioeconomic conditions (level of education and job). A total of 1482 children were examined and data associated with different socioeconomic parameters were determined in 763 of the subjects. The sample size was estimated according to prior studies (calculated as *P* = 0.05, confidence coefficient 99%). The inclusion criteria were 3 years of age and full eruption of deciduous teeth (20 teeth). Children with conditions affecting body weight or tooth decay, including diabetes, hyperthyroidism, hypothyroidism, malignancies, and renal diseases, and those who had permanent teeth other than the first molars in the oral cavity were excluded from the study.

The list of the centers eligible to be included in the study was provided by the Department of General Education and the Organization of Welfare and Public Health in Kerman. Cluster sampling method was used to select kindergartens and preschool centers from the list available. Before any clinical examinations, consent was obtained from the parents to include their children in the study. The examination was carried out one hour after breakfast, which included a piece of cheese, a slice of bread, and a cup of milk. Children's heights were determined in meter using a Laika (Taiwan) wall measuring tape. A Microlife digital weighting machine (Japan) was used to determine children's weight while they were wearing light cloths without any shoes. Body max index (BMI) was calculated using this formula:

(1) $$\begin{array}{c} \;\text{BMI}=\frac{\text{mass}\left(\text{Kg}\right)}{\left[\text{height}\left(\text{m}\right)\right]2}. \end{array}$$

A special form was completed for each child, which included personal data, age, gender, height, weight, and phone number. In subjects in whom the socioeconomic status was evaluated, the educational status of parents, parents' occupations, and the number of family members were also recorded in the form. BMI percentile was determined using standard tables based on age and gender. BMI was classified as follows [5, 7, 15]:

1. BMI percentile of <5 for age: weight under normal,
2. BMI percentile of ≥5 or <85: normal,
3. BMI percentile of ≥85 or <95: at risk for obesity,
4. BMI percentile >95: obese.

In the next stage, the teeth were examined for caries, restorations, and extractions based on dmft using a disposable dental mirror under 100 W light based on criteria proposed by the WHO. A postgraduate student of pediatric dentistry, who had been trained for one week in the Department of Pediatric Dentistry in the Faculty by a trained assistant before examination, carried out all the procedural steps and calculations. The examiner was tested until intraexamination error decreased. Based on the study protocol, the WHO definition was used to diagnose caries and in determining decayed teeth (dt) the following clinical forms were considered carious lesion: white spots, a discolored area or an area with a rough surface; teeth with temporary dressings; and teeth with any form of restorative material with signs of recurrent carries [15, 16]. Frequencies and percentages of categorical variables were reported. In addition, the mean ± SD for dmft index and its components according to our variables were reported. Poisson's regression model was used to evaluate the effect of BMI and various socioeconomic variables on dmft. Poisson's regression is appropriate for modeling counts of observations like dmft. A backward stepwise procedure was used to exclude independent variables from the adjusted model. The final model contained only factors that were significant at the level *P* ≤ 0.05. The rate ratios (RR) and 95% confidence intervals were reported in relation to Poisson's model corresponding to dmft index. Data were analyzed using SPSS 16.

## 3. Results

This study was a cross-sectional study. Of 1482 children evaluated, 257 (17.4%) were in the 3-4 age range, 451 (30.4%) were in the 4-5 age range, and 774 (52.2%) were in the 5-6 age range (Table 1).  Table 2 presents dmft and BMI values along with the three* d*,* m,* and * f* components of dmft separately based on the age groups. Poisson's regression model showed that after adjustment for age, BMI had a significant effect on children's dmft (*P* = 0.0001), with an inverse relationship between these two variables. On the whole, the mean of dmft for children with normal BMI was 1.5-fold higher than that in children who were overweight. In addition, age had a significant effect on dmft (*P* = 0.0001). In this context, with a one-year increase in age, the mean of dmft was 1.3-fold higher (Table 2). Various socioeconomic variables were evaluated in only 763 subjects because the parents had not provided answers to some questions. In the classification of BMI of the subjects in three groups, there were no subjects in the group with weight under normal; 83.8% subjects were in the group with normal weight and 12.1% subjects were in the overweight group (BMI percentile more than 85) and 4.1% had BMI percentile more than 95. Poisson's regression model did not reveal any significant effect of birth rank (*P* = 0.7), mothers' educational status (*P* = 0.26), and fathers' occupation (*P* = 0.5) on children's dmft. However, some other variables, including fathers' educational status and mothers' occupation, had a significant effect on children's dmft (Table 3). In addition, the effect of gender on dmft was significant (*P* = 0.0001). In this context, the mean of dmft in boys was 1.19-fold that in girls (Table 3). The prevalence of caries-free children in the present study was 38.3% and there were more caries-free children in the overweight children (41.2%). There were no significant relationships between BMI and parents' occupation status.

## 4. Discussion

The incidence of tooth decay is on the increase among Iranian children and adolescents. Azizi et al. reported a prevalence of 20.8% for obesity in 3–9-year-old children in Iran based on BMI. In addition, the prevalence of dmft in 3–9-year-old Iranian children was reported to be 1.9–3.6 [17]. The relationship between weight and tooth decay has always been a controversial issue. It is probable that intake of sugary foods might simultaneously result in weight gain and an increase in caries risk. Of course, when a low-sugar diet is consumed, BMI returns to normal but tooth decay continues, which is a factor that has resulted in identification of a significant relationship between DMFT and BMI in some studies. No doubt DMT/dmt score (carious and lost teeth) is calculated throughout life by considering all the teeth, but BMI is determined for each individual in a cross-sectional manner at a specific time interval. However a potential limitation of this cross-sectional study is that no cause-effect relationship can be deduced. In addition, it should be pointed out that the majority of studies have been retrospective studies carried out simultaneously in different communities with different cultures. Considering the detrimental effects of obesity during adulthood and its importance, the present study was undertaken to evaluate the relationship between BMI and tooth decay in 3–6-year-old children in the kindergartens and preschool centers in Kerman during 2009-2010.

The results showed a significant effect of BMI on children's dmft, with an inverse relationship between these two variables, consistent with the results of different studies carried out [7, 12, 18–20]. In contrast, some researchers have reported a direct relationship between BMI and dmft [11, 13, 21–25]. In other words, they reported that children's dmft increases with an increase in BMI.

The reasons for the discrepancies between the results of various studies might be evaluated from different aspects as follows.

*Subjects' Age.* The results of the present study showed that age has a significant effect on dmft; in other words, the mean dmft of children increases 1.5 times with a one-year increase in age: the mean of dmft increases by 32% with one-year increase in age. The majority of these studies have been carried out on subjects over 6 years of age [6, 11–14, 16, 22, 25, 26]. However, the present study was carried out on children aged 3 to 6. Growth and weight changes in children occur rapidly at this age and obesity can rapidly take place after changes in the diet in children this age. Therefore, if a child has tooth decay, the diet is one of the etiological factors.

The recent international growth reference, The WHO Child Growth Standards Reference, is used to evaluate nutritional status. This growth reference is recommended for young children from birth to 5 years and provides a scientifically reliable yardstick of children's growth achieved under desirable health and nutritional conditions and establishes the breastfed infant as the normative model against which all alternative feeding methods must be measured in terms of growth, health, and development; however it was not recommended for the present study due to children's age [27].

*Tooth Decay and Obesity*. Tooth decay and obesity are both considered multifactorial entities with predisposing genetic and environmental conditions and it is not possible to designate them as separate diseased conditions. In addition to the diet, some of the confounding factors involved in tooth decay are oral microflora, age, use of fluoride, and socioeconomic status. The effects of all these factors have not been evaluated in any study to date. Children who are underweight are more susceptible to infectious diseases such as tooth decay due to compromised immune system. In addition, families with children who are prone to obesity take greater care of their children's diet and provide them with less sweets and desserts, resulting in a low-caries prevalence in these children. The results of a study in Turkey (2011) showed that BMI increases in underweight children after receiving dental services [28]. Although obesity has been associated with factors such as the intake of fast-cooking foods and tooth decay has been associated with excessive intake of sweets, the real etiologies have not been definitely identified because the etiologies are complex and multifactorial. A large number of factors involved in obesity and tooth decay have originated from changes in life styles and environmental factors such as changes in the patterns of physical activities and the nutritional services offered in schools [10–13, 29, 30].

The results of the present study in subjects whose socioeconomic conditions were available showed that variables such as father's educational status and mother's occupation had a significant effect on children's dmft. Mother's occupation significantly altered caries status and children of mothers with better occupations experienced less caries; comparable results have been observed in previous studies [31–33]. Father's educational status significantly influenced the caries experience. A review of risk factors for dental caries in young children associated low father's and mother's education with high caries prevalence, similar to a study by Luepker [29].

Marshall et al. (2007) reported that low socioeconomic status results in an increase in the incidence of caries and obesity. In addition, the results of a study by Cinar and Murtomaa (2011) in Turkey showed that higher dmft rates in children are associated with the parents' low socioeconomic status [13, 28–35]. Researchers believe that there is no significant relationship between BMI and the family's socioeconomic status because in higher socioeconomic status a high BMI might be due to a high intake of proteins and in lower socioeconomic status high BMI might be the result of high carbohydrate intake. In the present study the mean of dmft in boys was higher than that in girls. In this context, the mean of dmft in boys was 1.19-fold that in girls. The prevalence of caries-free children in the present study was 37.1% and more caries-free children were found in the obese group, consistent with the results of a study by Sadeghi and Alizade in 2007 [15].

## 5. Conclusion

The present study showed that caries rate in the deciduous teeth of 3–6-year-old children decreases with an increase in body weight. On the whole, the mean of dmft for children with normal BMI was 1.5-fold that of children who were overweight.

Since tooth decay and obesity are multifactorial conditions and their relationship with individuals' nutritional habits has been established, the triad of sugar, tooth decay, and obesity should be evaluated in various studies. Therefore, despite an inverse relationship between tooth decay and BMI in the present study, prevention of tooth decay and obesity should be considered more than ever; in addition, the 3-day analysis of the diet in children should be considered in future studies. And also, no cause–effect relationship can be deduced from a cross-sectional design study such as this one. Longitudinal designs would increase the knowledge on the determinants of dental caries.

## Figures and Tables

**Table 1:** The demographic variables of children and their parents.

Variables		Boy		Girl		Total	
		*N*	*P*	*N*	*P*	*N*	*P*
		734	49.5	748	50.5	1482	100
Mother's education	Diploma and under diploma	264	56.4	204	43.6	468	100
	University	470	46.3	544	53.7	1014	100

Father's education	Diploma and under diploma	278	48.6	263	51.4	541	100
	University	456	48.4	485	51.6	941	100

**Table 2:** Distribution of dmft index and its various components along with BMI based on child age.

Age	Index					
		*d*	*m*	*f*	dmft	BMI
3 years	Mean ± SD Min–Max	1.47 ± 2.27 0–12	0.093 ± 0.529 0–4	0.16 ± 0.73 0–8	1.73 ± 2.65 0–13	15.4 ± 1.52 10.76–24.38

4 years	Mean ± SD Min–Max	1.91 ± 2.66 0–15	0.12 ± 0.64 0–6	0.29 ± 1.0 0–8	2.31 ± 3.09 0–16	15.41 ± 1.69 10.24–24.04

5 years	Mean ± SD Min–Max	2.25 ± 2.72 0–13	0.17 ± 0.65 0–7	0.57 ± 1.32 0–8	2.99 ± 3.25 0–14	15.5 ± 1.87 9.76–24.57

**Table 3:** Multiple Poisson regression analysis for effective factors on dmft index.

Variables		Adjusted rate ratio^1^	95% CI^2^	*P* value
Gender	Boy Girl	1.321 Reference	1.202–1.452 —	0.0001

BMI	Normal Overweight	1.449 Reference	1.270–1.653 —	0.0001

Father's education	Diploma and under diploma University	1.381 Reference	1.257–1.517	0.0001

Age		1.324	1.260–1.392	0.0001

Mother's job	Employee self-employment	1.347 Reference	1.117–1.624	0.0001

^1^The dmft ratio of each group versus reference group for each variable adjusted for other variables.

^2^Confidence interval.
